# Feasibility of Ultra-High Resolution Supra-Aortic CT Angiography: An Assessment of Diagnostic Image Quality and Radiation Dose

**DOI:** 10.3390/tomography7040059

**Published:** 2021-11-01

**Authors:** Felix Anton Ucar, Marius Frenzel, Mario Alberto Abello Mercado, Sebastian Altmann, Sebastian Reder, Carolin Brockmann, Marc A Brockmann, Ahmed E Othman

**Affiliations:** Department of Neuroradiology, University Medical Center Mainz, Langenbeckstrasse 1, 55101 Mainz, Germany; felix.ucar@web.de (F.A.U.); mariusafrenzel@gmail.com (M.F.); mario.abello@unimedizin-mainz.de (M.A.A.M.); sebastian.altmann@unimedizin-mainz.de (S.A.); sebastian.reder@unimedizin-mainz.de (S.R.); carolin.brockmann@unimedizin-mainz.de (C.B.); Marc.Brockmann@unimedizin-mainz.de (M.A.B.)

**Keywords:** computed tomography, computed tomography angiography, cerebral arteries, stroke, contrast media, image enhancement, radiation dosage

## Abstract

(1) *Background*: To evaluate diagnostic image quality and radiation exposure of ultra-high resolution cerebral Computed-Tomography (CT) angiography (CTA) obtained on an ultra-high resolution computed tomography scanner (UHR-CT). (2) *Methods*: Fifty consecutive patients with UHR-CTA were enrolled. Image reconstruction was processed with a 1024 × 1024 matrix and a slice thickness of 0.25 mm. Quantitative analyses comprising CT values, contrast–noise ratio (CNR) and signal-to-noise ratio (SNR) were performed. Subjective assessment of image quality, vessel contrast, noise, artefacts and delineation of different sized vessels were assessed by two readers on a 4-point scale. Radiation exposure was determined. (3) *Results*: Hounsfield values (ACI: 461.8 ± 16.8 HU; MCA: 406.1 ± 24.2 HU; BA: 412.2 ± 22.3 HU), SNR (ACI: 35.4 ± 13.1; MCA: 20.8 ± 12.4; BA: 23.7 ± 12.9) and CNR (ACI: 48.7 ± 21; MCA: 63.9 ± 26.9; BA: 48.1 ± 21.4) were remarkably high in all segments. Subjective analysis by two raters (fair agreement, k = 0.26) indicated excellent image qualities (image quality = 4; contrast = 4; noise = 3; artefacts = 4).Our analysis revealed a notably high traceability of the cerebral perforators (3 Points). Radiation exposure was at moderate dose levels (effective dose = 2.5 ± 0.6mSv). (4) *Conclusions*: UHR-CTA generates highly valuable image qualities that allow the depiction of vessels including cerebral perforators at acceptable dose levels. The UHR-CTA may therefore enhance the detection of small cerebral pathologies and may improve interpretability, especially in settings where high image qualities are crucial for the diagnostic accuracy.

## 1. Introduction

Computed tomography (CT) is an essential tool in neuroradiological imaging that has distinguished itself as quickly accessible and with an increasing level of diagnostic significance in clinical routine. Particularly in vascular imaging, noninvasive techniques have become highly relevant and, nowadays, CT represents a key component therein [1].

Nevertheless, clinically significant neurovascular pathologies such as aneurysms, arterial occlusions, vasculitic lesions [2], cerebrovascular malformations [3], vasospasms [4] and dissection can be particularly small and difficult to detect, and, thus, may be missed on standard CTAs [5]. The evaluation of these lesions can be additionally aggravated by Hounsfield artefacts of the posterior fossa due to the high density of the skull base and the petrous bone [6]. Hence, underlying vascular pathologies might evade the current resolution of standard CT systems or might not be interpreted sufficiently. These circumstances may involve follow-up examinations with additional radiation exposures as well as expenditure of time [7].The latter, however, is of prognostic relevance in acute neuroradiological emergencies such as ischemic stroke and hemorrhage. Depending on the type of the lesion and the size of the affected vessels, the evaluability of vascular alterations would be remarkably improved by an increasing resolution and image quality.

Recently, a UHR-CT, with a focal spot size of 0.4 mm × 0.5 mm, detector elements with 0.25 × 0.25 mm, a beam collimation of 0.25 mm × 160 mm rows and a slice thickness of 0.25 mm, has been introduced for clinical routine, enabling the acquisition of very high spatial resolutions up to 150 µm. This technological innovation has the potential to refine the detection of small pathologies that usually remain undetected on CT images with a conventional spatial resolution. We therefore investigated the quantitative and qualitative image quality and radiation dose of the supra-aortic UHR-CT angiography as a novel diagnostic tool to ameliorate neurovascular imaging, with a focus on the depiction of the cerebral vasculature including very small vessel branches.

## 2. Materials and Methods

### 2.1. Patients

This retrospective study was approved by the local Ethics Committee, and written informed consent was waived. All methods were carried out in accordance with relevant guidelines and regulations.

In total, 55 consecutive patients (23 men, 32 women, mean age 70.5 years; age range: 37–88 years) who underwent supra-aortic UHR-CTA in our institution were recruited. Of them, five patients did not meet the inclusion criteria due to poor image qualities (beam hardening artefact, distinct motion artefacts) with insufficient intra-arterial contrast, or in other cases, patients could not receive a bolus of contrast medium at a flow rate of 5 mL per second (4 women, 1 man, mean age 60; ranging from 23–81 years). A neuroradiologist with 10 years of experience assessed the sufficient arterial contrast required for inclusion in the cohort. A final number of 50 participants were subjected to our analysis (*n* = 50; 22 men, 28 women; mean age 70.5 years; age range: 37–88 years).

### 2.2. UHRCT System and CT Examination

CT images were acquired using the ultra-high resolution mode of the CE-certified UHR-CT Scanner (Aquilion Precision, Canon Medical Systems, Otawara, Japan) with the following specifications: focal spot 0.4 mm × 0.5 mm, detector elements with 0.25 × 0.25 mm and a beam collimation of 0.25 mm × 160 mm rows. By using these settings, images with a slice thickness of 0.25 mm were provided that were supposed to have an increased resolution in x–y axis and z-axis (150 µm).

CT angiography implied a focal spot size of 0.8 × 1.3 mm, a detailed pitch of 0.69, tube voltage of 120 kV, a field of view 180 mm and a rotation time of 0.5 s per rotation. Tube current was determined by auto exposure control (AEC) according to the individual patient. The underlying calibration for the dose length product (DLP) was carried out using a 32 cm test specimen (body).The helical scan was performed in caudo-cranial direction from the aortic arch to cranial vertex.

Contrast delivery was achieved by a double syringe system for advanced clinical CT imaging procedures (Accutron CT-D, Medtron, Saarbrücken, Germany).A nonionic contrast agent (Ultravist−370, Bayer Healthcare, Leverkusen, Germany) was given through a peripheral venous catheter (18 gauge plastic catheter) placed in the cubital vein. A total of 60 mL (370 mg iodine /mL) was injected with a flow rate of 5 mL/s and followed by a bolus of 60 mL isotonic saline administration (flow rate 5 mL/s). Routinely, a monitoring region of interest (ROI) was placed in the aortic arch to maximize scan start efficiency and simultaneously minimize the venous contamination. Timing was optimized using automatic trigger acquisition and enhancement threshold selection. After having reached a threshold of 180 Hounsfield units (HU) in the aortic arch, CTA acquisition started.

### 2.3. Post Processing and Image Reconstruction

CTAs axial raw data were reconstructed using a slice thickness of 0.25 mm, 1 mm and 3 mm from a pixel matrix of 1024 × 1024 and an iterative three-dimensional dose reduction algorithm (AIDR 3D standard, Canon Medical Systems, Otawara, Japan) together with a FC-41 kernel adapted for ultra-high resolution.

### 2.4. Radiation Dose

In this study, we intended to explore the estimated radiation dose descriptors including computed tomography dose index (CTDIvol) and DLP as reported by the CT system. Additionally, we estimated the effective dose, multiplying the dose length product by the conversion factor 0.0059 [8,9].

### 2.5. Quantitative Evaluation

In order to obtain objective parameters for UHR-image qualities, we measured and quantified HU values, signal-to-noise ratio (SNR) and contrast-to-noise ratio (CNR). The spatial resolution as well as the signal and the noise texture variation were kept constant and, therefore, CNR was used to define image quality and contrast.

To determine extracranial SNRs and CNRs, circular ROIs were drawn in the extracranial internal carotid artery (ACI) immediately after the carotid bifurcation, with a mean diameter value of 5.36 mm. The extravascular reference was measured using an ROI in the sternocleidomastoid muscle at exactly the same level and size. Circular ROIs were drawn in the mid-M1 middle cerebral artery (MCA) and in the mid-segment of the basilar artery (BA) with a mean diameter of 1.65 mm (MCA) and 2 mm (BA), respectively. The extravascular measurement was performed immediately adjacent to the vessel contour in the surrounding brain parenchyma by using the same ROI diameter as the vessel ROI. In order to prevent partial volume effects, ROIs were positioned centrally in the vessel lumen at maximum possible size, while avoiding the inclusion of the vessel wall or of atherosclerotic plaques. In the presence of hemodynamically relevant distal stenosis or vessel occlusions, the contralateral side was measured. Image noise was defined as the standard deviation of the intravasal measurements. SNR was calculated by dividing the CT density of the intravascular measurements (ACI, MCA and BA) by the image noise; CNR was calculated as follows: the intravascular CT density subtracted from the extravascular CT density, divided by the image noise.

### 2.6. Qualitative Evaluation

Subjective image quality was assessed by two radiologists (S.A; M.A; both with 5 years of experience in neurovascular imaging). Raters were briefed and trained with ten randomly selected patients not included in the study to attain a consensus and standardization on how to apply the 4-point Likert-like scale prior to image quality assessment. The scale was consistently used on all study participants and axial images with a slice thickness of 0.25 mm were evaluated. Multiplane reconstructions of 1 mm or 3 mm slice thickness, as well as maximum intensity projections and volume rendering projections.

Regarding the subjective image quality quantification, we focused on contrast and on the delineation of cerebral vessels of different sizes, general image quality, general vessel contrast, image noise, artefacts within the posterior fossa and, particularly, on the very small perforating cerebral arteries. The common carotid artery, the internal and external carotid arteries, the two vertebral arteries (representing large vessels) and the middle cerebral artery, the anterior cerebral artery and the basilar artery (representing medium sized vessels) were defined and assessed. The arteries supplying the cerebellum (posterior inferior cerebellar artery (PICA), anterior inferior cerebellar artery (AICA) and superior cerebellar artery (SCA), and the ophthalmic artery representing small arteries were also defined. In the perforator score described below, we focused on the lenticulostriate arteries arising from the M1 segment, pontine arteries and the thalamoperforating arteries.

Vascular contrast was assessed using the following 4-point scale: 4 = excellent contrast throughout all vessel sections; 3 = good contrast, not compromising diagnostic assessment; 2 = average contrast; 1 = poor contrast. Vascular delineation was assessed by applying the following: 4 = excellent delineation; 3 = good delineation, not compromising diagnostic assessment; 2 = average delineation, compromising diagnostic assessment, 1 = poor delineation. Notably, the evaluation of the general picture quality implied the spatial resolution, sharpness, contrast, image noise and artefacts and was composed as follows: 4 = Excellent; 3 = above average; 2 = average; 1 = poor. Classification of the general vascular contrast was defined as follows: 4 = Excellent with homogeneous distribution of the contrast agent and filling of the vascular course; 3 = good distribution of the contrast agent and contrasting of the vascular course; 2 = Partially inhomogeneous distribution and contrasting of the vascular course; 1 = poor distribution of the contrast agent and contrasting of the vascular course. The image noise was evaluated with the help of the following score: 4 = Excellent, with sharp vessel wall and well-delineated vessel contours due to poor image noise; 3 = very good, with well-preserved vessel wall definition and well-preserved delineated contour due to minimal image noise; 2 = good, with minimal limitations in the vessel wall/lumen definition due to moderate image noise; 1 = evident limitations in the vessel/wall/lumen definition due to strong image noise. Furthermore, the extent and influence of intracranial artefacts, especially streaks and dark bands in the posterior fossa were assessed. Artefacts were scored as follows: 4 = no artefacts; 3= minimal artifacts; 2 = artefacts of no diagnostic influence; 1 = artefacts with diagnostic consequence. Moreover, we defined and applied a score for the small cerebral perforator vessels: 4 = excellent visibility and traceability of the small perforators; 3 = good visibility and traceability of the small perforators; 2 = low visibility and significantly difficult traceability of the small perforators; 1 = small perforators not visible or traceable.

### 2.7. Statistical Analysis

Mean, median and standard deviation and interrater reliability for continuous variables were calculated (Cohen’s kappa coefficient) and determined using SPSS (IBM SPSS Statistics for Windows, Version 23.0. IBM Corp, Armonk, NY, USA).

## 3. Results

### 3.1. Quantitative Evaluation

**Figure 1:**
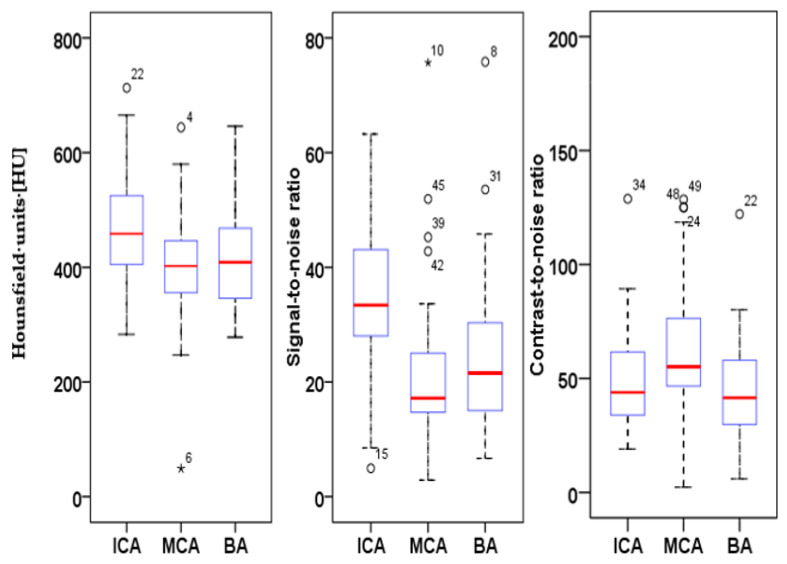
Boxplot of HU, SNR and CNR of the quantitative analysis of the internal carotid artery (ICA), middle cerebral artery (MCA) and basilar artery (BA) of 50 UHR-CTA images.

HU, SNR and CNR were calculated for three types of arteries (ICA, MCA and BA) in each patient, and value distribution was analyzed using a boxplot graph (Figure 1). Intravascular HU values averaged at 461.8 ± 16.8 HU for the internal carotid artery; 406.1 ± 24.2 HU for the middle cerebral artery and 412.2 ± 22.3 HU for the basilar artery (Figure 1). The SNR. e.g., ACI: 35.44 ± 13.1; MCA: 20.8 ± 12.4; BA 23.7 ± 12.9 and CNR, e.g., ACI: 48.7 ± 21; MCA: 63.9 ± 26.9; BA: 48.1 ± 21.4 of the obtained images was high in all analyzed vessels calculated from these values and confirmed our hypothesis about the improvement in vessel imaging (Figure 1).

### 3.2. Qualitative Evaluation

In addition to our quantitative analysis, a subjective image quality assessment was carried out using the set of parameters and the scoring matrix as described in Section 2.6. The results are listed in Table 1.

**Figure 2:**
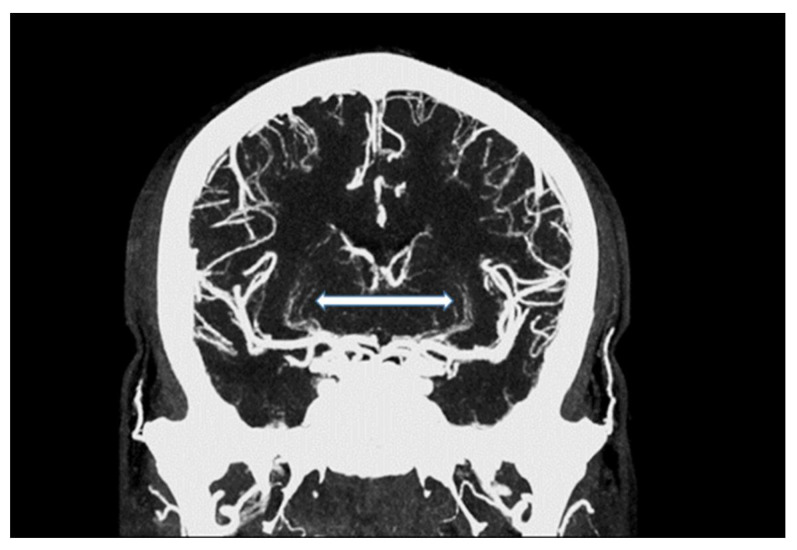
Maximum intensity projection of an intracranial UHR-CTA, depicting traceability of lenticulostriate arteries (LSA; white arrow).

**Figure 3:**
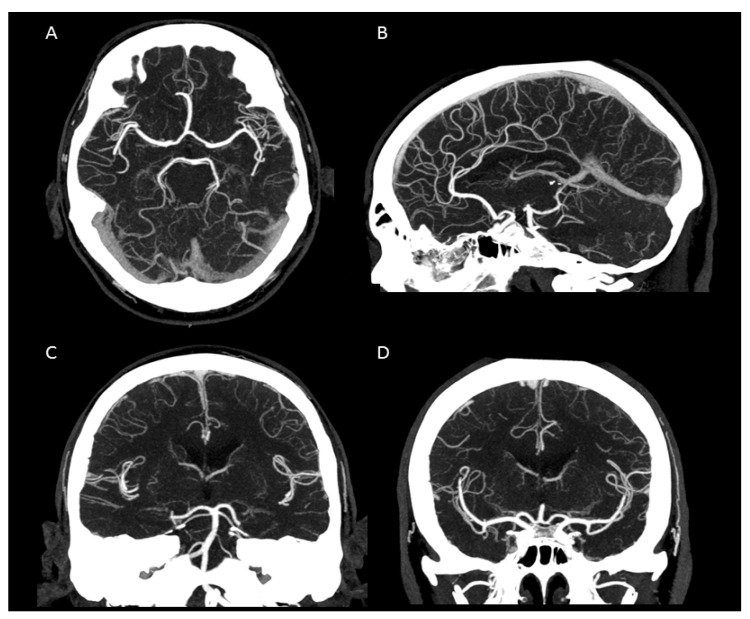
Maximum intensity projection of an intracranial UHR-CTA. (**A**) axial plane. (**B**) sagittal plane. (**C**,**D**) coronal plane. Note the clear traceability/visualization of the circle of Willis and even the small peripheral vessel branches.

**Figure 4:**
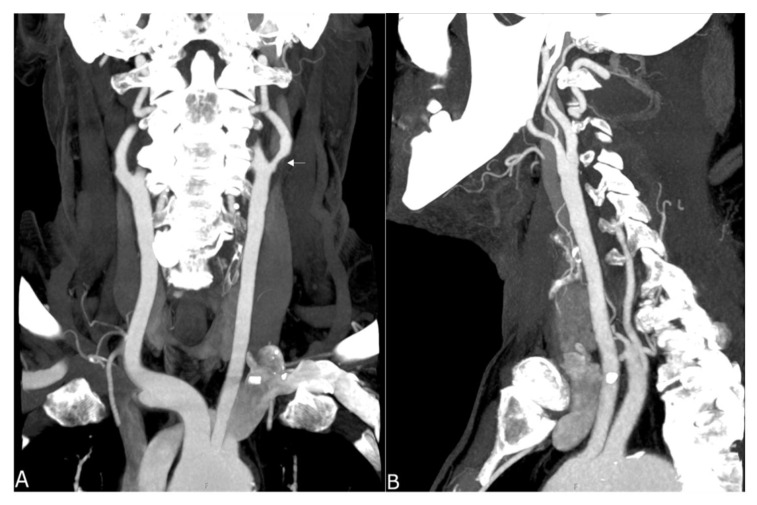
Maximum intensity projection of a supra-aortic UHR-CTA, (**A**) coronal projection showing an internal carotid artery plaque (white arrow) as well as contrast and delineation of, e.g., the thyrocervical trunk; (**B**) sagittal projection.

**Figure 5:**
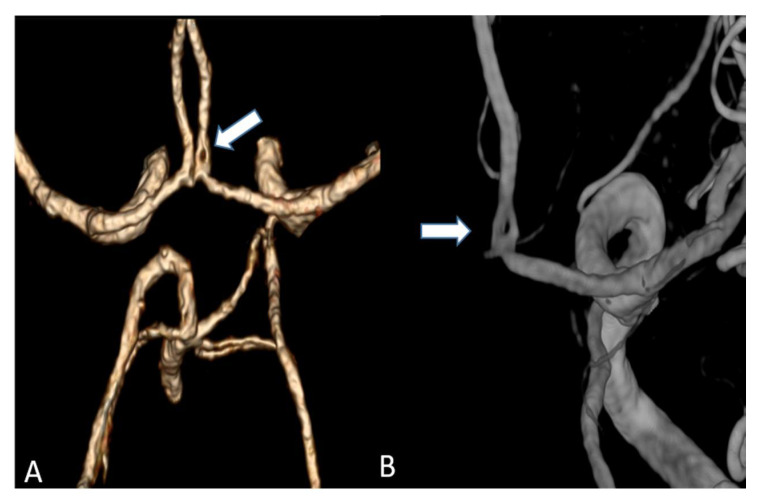
(**A**) Volume rendering of an anterior cerebral artery fenestration acquired by an UHR-CT (**B**) Three-dimensional (3D) digital subtraction angiography (DSA) to confirm arterial fenestration in (**A**), which is often not detected by normal resolution CTAs.

In summary, the subjective analysis of all images showed excellent results for the applied qualitative parameters such as overall image quality, overall contrast noise and artefacts, the latter focusing on streaks within the posterior fossa. A high-rated vessel contrast and delineation could also be observed, which were not only limited to the large cerebral vessels but also appeared in the smaller vessel sections, especially in the deep cerebral perforators (e.g., lenticulostriate arteries). Patient examples are given in Figure 2, Figure 3, Figure 4 and Figure 5. Interrater reliability was ascertained by applying Cohen’s kappa coefficient and showed a fair agreement (k = 0.26).

### 3.3. Radiation Dose

To analyze whether the radiation dose did not exceed the conventional dosage of CTA, the radiation dose during the UHR-CTA was determined. Dose exposure was described by CTDIvol and DLP, which were recorded by the CT console. CTDIvol was averaged at 11.7 ± 2.6 mGy and DLP ranged from 245.2 mGy*cm to 608.4 mGy*cm with an average at 433.9 ± 100.9 mGy*cm, resulting in a mean effective dose of 2.5 ± 0.6 mSv.

## 4. Discussion

The purpose of our study was to assess the image quality and dose exposure of the UHR-CTA and to evaluate its potential perspectives for routine application.

We were able to show that excellent image qualities were generated even though radiation exposure could be kept at explicitly moderate levels [10]. In addition, the occurrence of Hounsfield artefacts that frequently hinder the assessment of the vertebrobasilar system and the brainstem were also considerably low, enabling an advanced image interpretability. Both quantitative and qualitative evaluation of the UHR-CTAs confirmed our hypothesis of a highly remarkable improvement in image quality and spatial resolution. In particular, a high vascular contrast and a distinctly defined demarcation of the extracranial as well as the intracranial vessels could be demonstrated. It is noteworthy that even the smallest perforating arteries (e.g., LSA, as shown in Figure 3) vascularizing the deep brain structures become diagnostically accessible by UHR-CTA, which is generally not the case when using conventional CTA.

Studies on coronary artery and small visceral artery CTAs [11,12] have recently shown that the UHR-CT system is highly suitable for a significant improvement in image quality, SNR, CNR and an enhanced vascular imaging. Our investigation, which to our best knowledge represents the first study to evaluate image quality, contrast and delineation of the supra-aortic vessels and the small perforating arteries, confirms the excellent qualification of the UHR-CTA as a diagnostic tool in radiological and vascular imaging. Hence, the use of UHR-CTA will be of high value in various clinical settings, especially when a high resolution is inevitable for an accurate diagnosis and optimized patient care. In detail, advances in the diagnosis of aneurysms and steno-occlusive diseases may be reached, especially when looking at aneurysm configuration, and peculiarities or irregularities of the of aneurysm wall. UHR diagnostics may additionally improve pre-invasive planning of subsequent digital subtraction angiographies and interventions, enabling earlier and more individualized treatment decisions. An additional benefit will arise from a substantially reduced radiation exposure due to the omission or the time reduction in follow-up examinations.

Based on the fact that stroke represents one of most frequent life threatening incidences, and therapy is constantly being subjected to the innovation of more efficient methodologies, UHR-CTA may be a good candidate for facilitating the visualization of peripheral pathologies (e.g., peripheral occlusions such as M2 and others) and render them diagnostically visible. Thereby, acute interventional treatments such as thrombectomy or intra-arterial thrombolysis [13,14,15] may be optimized and may contribute to disease mitigation. This study has limitations. First, the retrospective design is associated with a certain selection bias, and the small sample size prevents a pathology-focused assessment of the included cases. The lack of comparison with a normal resolution CT sets an additional limitation to this study. However, our assessments represent an initial evaluation of the diagnostic image quality of the novel UHR-CTA as a basis for future pathology-focused diagnostic accuracy and radiation dosage studies. Even though with limited informative value, our findings strongly suggest a highly valuable diagnostic potential of UHR-CTA, presenting with excellent qualitative and quantitative image qualities and concomitant moderate radiation doses. UHR-CTA is, therefore, likely to introduce new perspectives in neurovascular imaging.

## 5. Conclusions

UHR-CTA generates highly valuable image qualities that allow the depiction of cerebral vessels including cerebral perforators at acceptable dose levels. The UHR-CTA may, therefore, enhance the detection of small cerebral pathologies and may improve interpretability, especially in settings where high image qualities are crucial for the diagnostic accuracy.

## Figures and Tables

**Table 1:** Medians and interquartile range (IQR) of qualitative parameters of 50 UHR-CTA images rated by two trained readers (radiologists).

Assessed Parameter	Reader 1 (IQR)	Reader 2 (IQR)
Overall Quality	4 (3–4)	4 (3–4)
Overall Contrast	4 (4–4)	4 (4–4)
Noise	3 (3–4)	3 (3–4)
Artefacts	4 (4–4)	4 (4–4)
Contrast (large vessels)	4 (4–4)	4 (4–4)
Delineation (large vessels)	4 (4–4)	4 (3–4)
Contrast (medium vessels)	4 (4–4)	4 (3–4)
Delineation (medium vessels)	4 (4–4)	4 (3–4)
Contrast (small vessels)	4 (3–4)	4 (3–4)
Delineation (small vessels)	3 (3–4)	3 (3–4)
Visibility and traceability of perforators	3 (3–4)	4 (3–4)

## Data Availability

The data presented in this study are available on request from the corresponding author. The data are not publicly available due to ethical and data privacy regulations.

## Abbreviations

HU	Hounsfield unit
CT	computed tomography
UHR	ultra-high resolution
CTA	computed tomography angiography
CNR	Contrast-to-noise ratio
SNR	Signal-to-noise ratio
CTDIvol	computed tomography dose index
DLP	dose length product
MCA	middle cerebral artery
ACI	internal carotid artery
LSA	lenticulostriate arteries
SV	Sievert
Gy	Gray
ROI	region of interest
IQR	interquartile range
PICA	posterior inferior cerebellar artery
AICA	anterior inferior cerebellar artery
SCA	superior cerebellar artery
